# A systematic review of mental health in rural Andean populations in Latin America during the COVID-19 pandemic

**DOI:** 10.3389/fpsyt.2023.1136328

**Published:** 2023-08-17

**Authors:** Jeel Moya-Salazar, Alexis Jaime-Quispe, Betsy Cañari, Jeel G. Moya-Espinoza, Hans Contreras-Pulache

**Affiliations:** ^1^Faculties of Health Science, Universidad Privada del Norte, Lima, Peru; ^2^South America Center for Education and Research in Public Health, Universidad Norbert Wiener, Lima, Peru; ^3^Qualitative Unit, Nesh Hubbs, Lima, Peru

**Keywords:** COVID-19, mental health, Latin America, depression, rural population

## Abstract

**Background:**

COVID-19 has been causing mental health problems around the world, with rural and indigenous peoples likely to be the most affected. This systematic review synthesizes and critically analyzes the existing literature on mental disorders in the rural Andean population in Latin America.

**Methods:**

A systematic review with narrative synthesis was carried out following the PRISMA guidelines. We searched nine databases (PubMed/MEDLINE, Scopus, EMBASE, ScienceDirect, Web of Science, Cochrane, Scielo, LILACS, and Latindex), five public prepublication servers (SocArXiv, medRxiv, bioRxiv, SportRXiv, and Preprints), ALICIA, and Google Scholar for articles that included the analysis of mental health problems using data collected from the rural Andean population in Latin America. These were eligible for inclusion. Articles that included Non-Latin American populations (including European or African migrants) and studies conducted prior to the COVID-19 pandemic (since the declaration of national lockdown) were excluded.

**Results:**

A total of 23,761 articles were retrieved, 14 of which met the inclusion requirements. Most were cross-sectional (*n* = 12) and related to anxiety (*n* = 9), depression (*n* = 8), and stress (*n* = 5). The mental health analysis of 5,976 rural dwellers from four countries in Latin America also included gray literature studies (*n* = 7) that allowed the quantification of mental health problems in adults (*n* = 7) and adolescents/children (*n* = 4). Only one study was multinational, and the quality of publications varied. Despite the high frequency of anxiety, depression, and stress symptoms among rural Latin American populations during COVID-19, published research is very limited. This review found preliminary evidence that the frequency of anxiety (45%), depression (27.6%), and stress (33.1%) in the rural population was associated with pandemic restrictions across countries. Measures of other psychiatric problems, such as distress or suicidal ideation, cannot be estimated.

**Conclusion:**

Regional-wide studies investigating changes in the frequency of symptoms of mental health problems in the context of the COVID-19 pandemic are warranted to inform culturally adapted prevention strategies. This study is limited to a narrative synthesis and may be subject to publication bias.

**Systematic review registration:**

https://www.crd.york.ac.uk/prospero/display_record.php?RecordID=320489.

## 1. Introduction

Disparities in mental health among urban and rural populations show a marked gap, which are related to a number of factors ranging from political to anthropological factors. This phenomenon is present in high-income as well as middle- and low-income (LMIC) populations. However, these differences are accentuated in countries with a great proportion of LMIC rural or peri-urban populations, which is a public health problem (1). COVID-19 has shown these differences and has led to more inequity with respect to access to mental health in rural populations (2). Previous studies have demonstrated that anxiety, depression, and suicide levels have been higher in rural populations in comparison with urban populations (3, 4). However, other studies have shown low levels of anxiety and depression in rural populations (5, 6).

The impact of mental health can vary between rural and urban populations due to a combination of factors. Economic characteristics, societal differences, and the specific effects of COVID-19, such as mortality rates and quarantine measures, can contribute to these differences. One significant factor is the limited access to mental health services in rural areas, where there is a scarcity of trained psychologists and psychiatrists and a lack of suitable facilities (7). Moreover, stigma surrounding mental health issues, social isolation, limited social support, socioeconomic conditions, lifestyles, and environmental factors can all play a role in influencing the wellbeing of rural communities (8). It has also been observed that the adoption of protective behaviors against COVID-19 and health literacy during the pandemic differ among different socioeconomic groups in Iran, with lower levels reported in populations of low socioeconomic status (9).

Thus, not all the rural communities have felt the pandemic in the same way, and the neuropsychological impact can vary (10). In general, communities in Latin America face regional challenges, social and political conflicts, and have high levels of violence that can lead to mental disorders (11, 12). Rural populations in Latin America are grouped in the Andes (distributed between Peru, Chile, Colombia, Ecuador, Argentina, and Bolivia), and they suffer inequities that are marked compared to urban populations, characterized by low human development, low level of access to healthcare, economic limitations, and social, religious and cultural issues (13). Hence, COVID-19 can have a kickback effect on rural communities' mental health, where these disruptions have not been quantified.

The objective of this systematic review was to estimate the mental health problems among rural Andean populations in Latin America during the COVID-19 pandemic, highlighting the differences among inter- and intra-population groups.

## 2. Materials and methods

### 2.1. Study design, search databases, and strategy

From 15 December 2021 to 2 January 2022, we searched nine databases (PubMed/MEDLINE, Scopus, EMBASE, ScienceDirect, Web of Science, Cochrane, Scielo, LILACS, and Latindex), five public prepublication servers (SocArXiv, medRxiv, bioRxiv, SportRXiv, and Preprints), a Peruvian thesis repository (ALICIA ConCyTec), and Google Scholar. These last two databases include gray literature, making the research search more extensive. This review follows the reporting guidelines specified in the Preferred Reporting Items for Systematic Reviews and Meta-Analysis (PRISMA) statement (14). This review was previously registered with PROSPERO (CRD42022320489).

The database search strategy was carried out using Boolean descriptors with a combination of keywords and subject headings. We identified publications using the terms (((Andes) OR (rural population [Mesh])) AND (mental health [Mesh])) AND (COVID-19 OR SARS-CoV-2 OR Pandemic) AND (Latin America))) and the corresponding Spanish and Portuguese translations. Manual searching was performed on the reference lists of included studies without filters or limits used when studies meeting the inclusion criteria were identified.

### 2.2. Inclusion and exclusion criteria

The included studies met the following criteria: (i) Latin American general population; (ii) studies that evaluate mental health problems; (iii) original studies (prospective or retrospective), clinical trials, case–control studies, perspectives, and scientific letters; (iv) articles in English, Portuguese, and Spanish; and (v) rural or indigenous populations of the Andes of Latin America. Narrative reviews, systematic reviews, meta-analyses, reflection articles, position papers, and letters to the editor (correspondence) were excluded. We also excluded non-Latin or urban American populations (including European or African migrants) and studies conducted prior to the COVID-19 pandemic (since the declaration of national lockdown due to the patient zero case report). We considered only studies from 2020 to 2021 related to the time of the pandemic in the region.

### 2.3. Screening study, data extraction, and quality assessment

**Figure 1:**
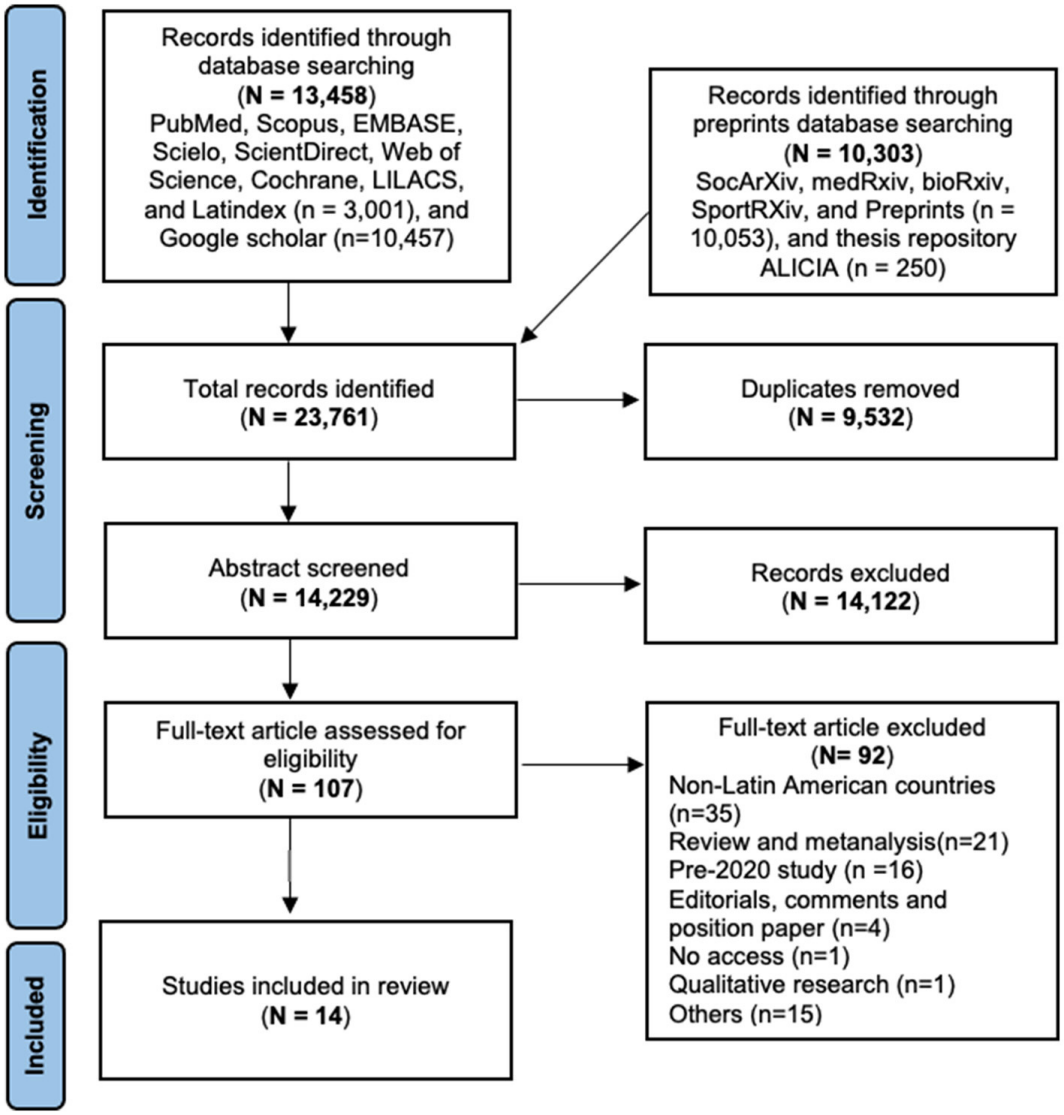
PRISMA flowchart for the selection of studies on mental health in the rural population.

Two independent authors (JM-S and AJ-Q) sifted the abstracts and excluded those that did not meet the inclusion criteria following the defined protocol. These authors also manually reviewed the full-text articles, and the disagreements were resolved by consensus at each stage of the revision (Figure 1). At each stage of the review process, meetings were conducted to ensure compatibility and consistency in the results of all measures. These meetings served as a platform to address any discrepancies or differences in the selection of articles, fostering consensus among the team members. Studies were grouped by country and type of mental illness (i.e., stress).

For the synthesis of the selected studies, we used the template of the Critical Appraisal Skills Programme (CASPe) group, which allowed us to select the information from each study (15). Furthermore, to ensure the validity of our findings, the risk of bias was independently assessed by both authors using the Cochrane risk of bias tool (16, 17). Studies that failed to report limitations or biases and inadequately described the rural Andean population were collectively deemed to have a high risk of confounding based on consensus. This rigorous evaluation of bias helped strengthen the reliability and integrity of our study results (Supplementary material).

### 2.4. Data analysis

A complete reading of each study was carried out, extracting the baseline characteristics of the studies (i.e., country and population), the instruments used (i.e., Beck's anxiety questionnaire), and the outcomes (i.e., association between depression and anxiety in the Colombian population). In this study, we used MS-Excel 2013 (Microsoft Corp., Redmond, Washington) and SPSS version 23.0 (IBM, Armonk) for data management (data for presentation or synthesis) and analysis, respectively. The collected data will allow us to compare at inter- and intrapopulation levels, which enables us to define the global frequency and the changes between countries. Regarding mental illnesses, studies from each country were carefully selected, and their respective averages for anxiety, depression, and stress measures were estimated. To provide a visual representation of these measures, Bing Excel Maps (Microsoft) were utilized, allowing for effective mapping and analysis of the data.

## 3. Results

### 3.1. Search results

The search in the databases yielded 23,761 records, of which we eliminated 9,532 duplicates. Then, we examined 14,229 abstracts and excluded 14,122, reviewing a total of 107 full-text articles. After we finished the revision, 92 studies were eliminated, mainly studies about mental health in non-Latin American populations (*n* = 35), which resulted in a total of 14 studies included for qualitative synthesis (Figure 1). According to the kappa analysis, the two independent reviewers had “substantial agreement” on the selection of abstracts (kappa = 0.81) and the full-text revision (kappa = 0.85) (18).

### 3.2. Characteristics of the studies

We performed an analysis of mental health in 5,976 rural dwellers from four countries of Latin America, in 14 studies, where Peru had the biggest proportion of participants (*n* = 2,359) (19–32). We also included a regional-wide study that analyzed 708 adults from Argentina, Chile, Colombia, Costa Rica, Ecuador, Guatemala, Panama, Paraguay, Peru, Uruguay, and Mexico (32). In addition, five studies from Peru (33.3%) (27–31), four studies from Ecuador (26.6%) (22–25), three from Colombia (20%) (19–21), and a study from Paraguay (26) were included. Of the total, 12 (80%) studies were cross-sectional (19–23, 25, 26, 28–32), two (13.3%) were cohort studies (17, 24), and one was a mixed study (24). On the assessed population, 10 (66.7%) studies included the adult population in rural areas (19–21, 24, 26, 28–30, 32), 4 (26.6%) included children and adolescents (22, 23, 25, 31), and 1 study included both populations (24) (Table 1).

**Table 1:** Baseline characteristics of the selected studies.

**Country**	**References**	**Study design**	***N* total**	**Population**	**City**
Colombia	Caballero-Domínguez et al. (19)	Cross-sectional	435	Adults	N/A
	Moya et al. (20)	Cross-sectional	576	Adults	Temuco
	Galvis and Güiza (21)	Cross-sectional	12	Adults	Bucaramanga
Ecuador	Cifuentes and Navas (22)	Cross-sectional	895	Children and adolescents	N/A
	Reategui (23)	Cross-sectional	60	Children	Juan Montalvo
	Guevara (24)	Mixed	27	Adults	Santa Elena
	Casa (25)	Cross-sectional	200	Children and adolescents	Cotopaxi
Paraguay	Torales et al. (26)	Cross-sectional	703	Adults	San Lorenzo
Peru	Porter et al. (27)	Cohort	1,911	Adults and adolescents	N/A
	Cieza et al. (28)	Cross-sectional	95	Adult	Ayacucho
	Ramos (29)	Cross-sectional	83	Adult	Arequipa
	Millones-Morales et al. (30)	Cross-sectional	115	Adults and older adults	Lima-Comas
	Santamaría (31)	Cross-sectional	155	Adolescents	Piura
Regional^*^	Durán-Agüero et al. (32)	Cross-sectional	708	Adults	...^**^

^*^This study includes the population from Argentina, Chile, Colombia, Costa Rica, Ecuador, Guatemala, Panama, Paraguay, Peru, Uruguay, and Mexico. ^**^Does not include the analyzed cities. Ref, References.

### 3.3. Mental health approach in rural population

In Colombia, we registered 3 studies that evaluated 1,023 rural adults, of which 2 studies estimated depression, stress, and anxiety (17, 18), while 1 assessed fatalism relating to COVID-19 associated with suicide (19). Four studies included the Ecuadorian population with 1,182 participants, of which only 1 study evaluated the adult population (24). In addition, emotional health (22), behavioral disorders (23), psychosocial impact due to tourism restrictions (24), and stress and anxiety in children and adolescents (25) were evaluated in the Ecuadorian population. In this revision, we included only one cross-sectional study in the Paraguayan adult population in which depression disorders were evaluated (26). On the other hand, five Peruvian studies were included in this revision, three analyzed the adult population (28–30), and a cross-sectional study included only adolescents (31) with a total population of 2,359 participants. Four Peruvian studies assessed anxiety, depression, or stress, and only one evaluated the role of poverty in the development of mental disorders (28). Finally, a regional-wide study with 10,552 participants included 435 rural adults from Argentina, Chile, Colombia, Costa Rica, Ecuador, Guatemala, Panama, Paraguay, Peru, Uruguay, and Mexico (18).

### 3.4. Anxiety, depression, and stress in rural population from Latin America

Nine studies addressed anxiety disorders in the rural population (Table 2). In the Colombian population, an increase in cases of anxiety during the pandemic was observed (20, 21), while studies in Ecuadorian children and adolescents estimated an average of 5.5% of anxiety (23, 25). Four studies assessed anxiety in Peruvian adolescents and adults. The levels of anxiety fluctuated between 26 and 95.7%, in which the study conducted by Millones-Morales and Gonzales-Guevara (30) reported that 52.2 and 15.7% of the aged had severe anxiety. Finally, a regional study by Durán-Agüero et al. (32), in 11 Latin American countries, reported levels of low, moderate, and severe anxiety in 23.4, 24.5, and 16.1%, respectively.

**Table 2:** Anxiety scenario in the rural population from Latin America.

**Country**	**References**	***N***	**Population**	**Instrument**	**Main outcome**
Colombia	Moya et al. (20)	803	Adults	SCL-90R	Anxiety increased to 22% with respect to pre-pandemic measurement (8.4%)
Colombia	Galvis and Güiza (21)	12	Adults	BAI	Anxiety in 100% of older adults; 75 and 25% with moderate and severe anxiety, respectively
Ecuador	Casa (25)	200	Children and adolescents	SCAS-Child	Low, moderate, and high levels of anxiety in 94.5, 4, and 1.5%, respectively
Ecuador	Reategui (23)	60	Children	EDAH	Anxiety in 8% of children
Peru	Porter et al. (27)	1911	Adults and adolescents	GAD-7	Anxiety in 32% (95% CI, 29.42–33.59)
Peru	Ramos (29)	83	Adults	CAS	Anxiety in 26% of cleaning workers
Peru	Millones-Morales and Gonzales-Guevara (30)	115	Adults and older adults	DASS-21	Anxiety in 95.7%: 52.2% very severe, 15.7% severe, 17.4% moderate, and 10.4% mild
Peru	Santamaría (31)	155	Adolescents	DASS-21	Anxiety in 55.5%: 16.8% very severe, 12.3% severe, 18.1% moderate, and 8.4% mild
Regional^*^	Durán-Agüero et al. (32)	708	Adults	BAI	Anxiety in 61%: 23.4% low, 24.5% moderate, and 16.1% severe anxiety

DASS-21, The Depression, Anxiety and Stress Scale-21 Items; GAD-7, General Anxiety Disorder-7; SCL-90R, Symptoms Checklist-90-Revised; BAI, Beck's Anxiety Inventory; SCAS-Child, Spence Children's Anxiety Scale; CAS, Coronavirus Anxiety Scale; Ref, References.

^*^This study includes population from Argentina, Chile, Colombia, Costa Rica, Ecuador, Guatemala, Panama, Paraguay, Peru, Uruguay, and Mexico.

Depression was reported in eight studies (Table 3). They have shown an increase in the levels of depression during the COVID-19 pandemic in the Colombian population, even though it was reported that 100% of the population did not have a depressive disorder. In Ecuador (25), between 5 and 11% of children and adults were reported to have depression, while 12.29% of the Paraguayan population (26) had some form of depressive disorder, including a mixed anxiety-depressive disorder (9.78%). In addition, 56.2% of the Peruvian rural population had depression during the COVID-19 pandemic. The study by Millones-Morales and Gonzales-Guevara (30) on the aged who live in rural areas has demonstrated that there exists 38.3, 9.3, and 27% of very severe, severe, and moderate depression, respectively.

**Table 3:** Studies that have reported levels of depression in the Latin American rural population.

**Country**	**References**	***N***	**Population**	**Instrument**	**Outcomes**
Colombia	Moya et al. (20)	803	Adults	SCL-90R	Anxiety increased to 24.1% with respect to pre-pandemic measurement (19%)
Colombia	Galvis and Güiza (21)	12	Adults	BDI	100% of older adults without depression
Ecuador	Guevara (24)	27	Adults	Own survey	Depression in 11% of the population
Ecuador	Reategui (23)	60	Children	CRS-R	Depression in 5% of children
Paraguay	Torales et al. (26)	703	Adults	Clinical record	Depression in 12.29%: mild depression in 16.3% and moderate depression in 23.9%; and mixed anxiety-depressive disorder in 9.78%
Peru	Porter et al. (27)	1,911	Adults and adolescents	PHQ-8	Depression in 27% (95%CI, 24.65–28.62) of adults
Peru	Millones-Morales and Gonzales-Guevara (30)	115	Adults and older adults	DASS-21	Depression in 91.3%: 38.3% very severe, 9.3% severe, 27% moderate, and 16.5% mild
Peru	Santamaría (31)	155	Adolescents	DASS-21	Depression in 50.3%: 10.3% very severe, 7.1% severe, 15.5% moderate, and 17.4% mild

PHQ-8, Patient Health Questionnaire Depression Scale; DASS-21, The Depression, Anxiety and Stress Scale-21 Items; SCL-90R, Symptoms Checklist-90-Revised; BDI, Beck's Depression Inventory; CRS-R, The Conners' Rating Scales-Revised; Ref, References.

Five studies reported the levels of stress in the rural population (Table 4). There has been an increase in stress levels during the COVID-19 pandemic in the Colombian population, which affects 36.9% of the population (30). Interestingly, 88.5% of Ecuadorian children and adolescents had low levels of stress in comparison with 19% of adults (24, 25). On average, in Peru, 48.75% of adolescents, adults, and older adults presented with levels of stress during the COVID-19 pandemic (30, 31).

**Table 4:** Studies that have reported levels of stress in the Latin American rural population.

**Country**	**References**	***N***	**Population**	**Instrument**	**Outcomes**
Colombia	Moya et al. (20)	803	Adults	PSI	Stress increased to 36.9% with respect to pre-pandemic measurement (27%)
Ecuador	Casa (25)	200	Children and adolescents	DISS	Low, moderate, and high levels of stress in 88.5, 7.5, and 4%, respectively
Ecuador	Guevara (24)	27	Adults	Own survey	Stress in 19% of the population
Peru	Millones-Morales and Gonzales-Guevara (30)	115	Adults and older adults	DASS-21	Stress in 65.2%: 16.5% very severe, 25.2% severe, 10.4% moderate, and 13% mild
Peru	Santamaría (31)	155	Adolescents	DASS-21	Stress in 32.3%: 3.9% very severe, 6.5% severe, 12.9% moderate, and 9% mild

PSI, Parenting Stress Index; DASS-21, The Depression, Anxiety and Stress Scale-21 Items; MBI-ES, Maslach Burnout Inventory-Educators Survey; DISS, Daily Infant Stress Scale; Ref, References.

**Figure 2:**
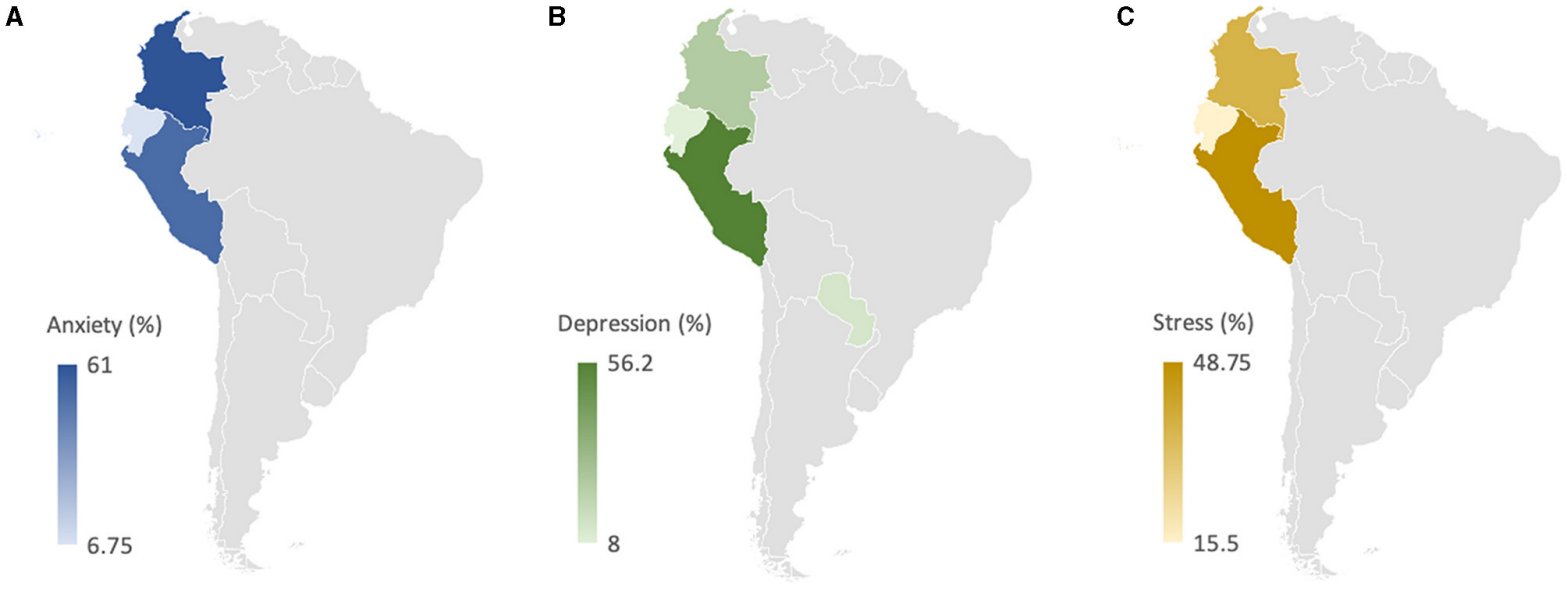
Average distribution of anxiety **(A)**, depression **(B)**, and stress **(C)** by Latin American country. Average anxiety among rural populations in Colombia, Peru, and Ecuador was 61, 52.3, and 6.75%, respectively. The average depression in the Peruvian population was 56.2%, in the Colombian rural population was 24.1%, in the Paraguayan population was 12.3%, and in the Ecuadorian population was 8%. The average stress in Peru, Colombia, and Ecuador was 48.7, 36.9, and 15.5%, respectively.

In Figure 2, the average distribution of the main mental health problems during the COVID-19 pandemic is shown; thus, the rural population with anxiety, depression, and stress was 45.08, 27.6, and 33.1%, respectively.

### 3.5. Other components of mental health in Latin America

Cifuentes and Navas (22) have demonstrated that 21.8% of Ecuadorian children and adolescents have reported mental disorders. Although, during the COVID-19 quarantine, good coexistence was reported with a high predominance of 52.7%, there were variables associated with mental health disorders such as rural origin, changes in the schedules of the tasks, a lack of physical exercise, and some emotions such as sadness, fear, anger, and joy. The study conducted by Reategui (23) has shown that rural children present with depression (5%), anxiety (8%), hyperactivity (12%), and attention deficit (35%) due to stressor factors (48%). In this population, 78% of the parents did not know about mental health, and 75% did not know where to go in case of mental disorders (23).

## 4. Discussion

This is the first systematic review of the literature on mental health problems in the Latin American population of rural areas of the Andes during the COVID-19 pandemic. We identified only 14 articles, which reveal that, in spite of the mental healthcare interventions during the pandemic, there exists an important frequency of symptoms of anxiety, depression, and stress in a limited group of publications on this important subject. Almost all of the articles were cross-sectional studies restricted to analyzing the adult or adolescent population of only one country. In addition, almost half of the documents included in this revision were gray literature from two-fourths of Latin American countries that had studies on rural populations in the Andes during the lockdown.

The main strength of this study is, for the first time, the analysis of mental health in the Andean rural population using gray and black literature. Two recent systematic revisions centered on LMICs have determined that Latin America, after Africa and Asia, has shown the worst overall mental health symptoms (33, 34). However, these meta-analyses have not included the rural or indigenous populations, which may have more deteriorated mental health. This even more deteriorated condition might be due to a mosaic of factors that include a lack of dialogue about sanitary decision-making in the face of COVID-19, which would already precede as a crucial factor (35), lack of access to quality healthcare services, low coverage and disorganization of medical attention (36), limited recourses to extend coverage of mental health programs, and low economic-social sustainability (6, 37).

According to World Bank data, 60% of the LMIC population lives in rural areas (India: 66%, China: 40%, and Sub-Saharan Africa: 59%) (38), communities where the pandemic has worsened in terms of infection and deaths. Other diseases such as the occurrence of cancer in the indigenous population are consequences of the disparities these regions face (39). In addition, the pandemic has accentuated this disparity due to the fact that several health programs are being abandoned or have been limited, which affects the rural population to a greater extent in comparison to the urban population concerning breast (86 vs. 88%) and cervical cancer (77 vs. 82%) screening (40). Taking this context into account, the rural population has been living in a paradox more than ever since health could not become sustainable with local needs, while confinement and the immediate cessation of their social and economic activities invaded their daily thoughts and caused mental conflicts.

Similar to our investigation, multiple studies informed us that mental health had deteriorated during the pandemic, which caused an increase in depression symptoms in Latin American countries (33, 34, 41). Furthermore, the prevalence of depression was reported to be 27% in the general European population (42), similar to our result in the rural population (27.6%). However, our results are different from the symptoms of depression reported in the general population of Southeast Asia (16%) and in Africa (45%) (43, 44). Our results suggested that approximately 45% of the rural population in Latin America has reported anxiety symptoms, showing a significant increase in previous reports in the general population worldwide (42–44). Social, economic, and cultural characteristics are key factors contributing to the different prevalence of depression and anxiety across regions and populations.

During the pandemic, indigenous and rural populations have highlighted their inequities while adopting health measures that have little dialogue with their social realities, even when knowledge about health and disease prevention is acquired through community interaction (5). As our results suggest, mental health programs do not appear to have an overall impact on the development of anxiety, depression, or stress symptoms in rural populations. In addition, it has been reported that the quality of life of rural and indigenous populations has been affected during the pandemic, so it is important to conduct organized activities to monitor mental issues in order to avoid complications, such as the high frequency of suicide in the rural populations (4).

Several studies have examined mental health on different continents, mainly focusing on urban populations and students. Systematic reviews conducted in Southeast Asian (43), African (44), Latin American (34), and Spanish (45) populations have revealed prevalence rates of anxiety symptoms ranging from 20 to 37%. These findings suggest a lower prevalence of anxiety compared to the results obtained in our study of the rural population. Similarly, our study indicated that 27.6% of the rural Latin American population exhibited symptoms of depression, which contrasts with the reported rates of 16% and 22% in Southeast Asian (43) and Spanish (45) populations, respectively. However, studies conducted in urban African (45%) and Latin American (35%) populations have reported higher prevalence rates (34, 44). These discrepancies may be attributed to the fact that the rural population is exposed to distinct social, economic, and political factors that can impact their mental health and potentially diminish their overall quality of life (8).

Language is an important factor to consider as non–Spanish-speaking populations have shown higher rates of mental health symptoms (34). In rural Latin American communities, where languages such as Quechua are commonly used, language can influence both the comprehension of COVID-19 prevention and control measures and the limited access to mental healthcare services provided in specific languages (7, 46, 47). Additionally, another influential factor is the prevalence of violence within Latin American populations (11, 47). Zhang et al. (34) study demonstrated a higher prevalence of anxiety in urban populations compared to Spanish (45) and Southeast Asian (43) populations. While this review supports our findings, our study indicates a greater burden of mental health problems in the rural population. These communities may face higher levels of violence, which may have been exacerbated during the pandemic, further worsening mental health (48–50). Additional research is needed to explore the role of these factors in mental health outcomes.

This study has limitations that must be acknowledged. First, the heterogeneity of the identified studies precluded meta-analysis, leading to the presentation of results in narrative form. Second, due to the nature of the lockdowns and social isolation during the pandemic in each country, there have been empirical and methodological limitations of each study (i.e., convenience sampling and various data collection tools) that have prevented oversimplification of the findings. Third, we included several gray literature studies that were limited by the varying quality of the documents identified (21–25, 29, 31). However, bias analysis of the studies has reported the risks of each study. Our study findings have revealed an important issue regarding the varying definitions of the rural population across different countries. This variation poses a risk of selection bias and misclassification, potentially hindering the generalizability of our interpopulation analysis. To address this limitation and ensure accurate characterization of the population in future research, it becomes imperative to stratify the population based on specific indicators. Fourth, mental health problems have been addressed differently in each study; thus, only some articles have reported levels of suicide or distress, and, therefore, the findings have not been generalized. Furthermore, most of the studies have been carried out in the adult rural population, but others have included adolescents and the elderly, where not all mental health issues have been evaluated (22, 23, 25, 27, 30, 31).

## 5. Conclusion

This is the first systematic review of mental health in the rural population of Latin America during the COVID-19 pandemic. The results identified adults, children, and adolescents with symptoms of anxiety, depression, and stress. Studies are limited and not available for all countries, and prospective designs are required to understand the changes in mental health problems in the epidemiological context of a health emergency and the subsequent sequelae once the pandemic is over. Further regional studies targeting indigenous and rural vulnerable groups are needed to determine the depth of mental illness and quality of life of populations, refine WHO guidelines, and inform the development of evidence-based and tailored mental illness prevention activities adapted to the culture of each population.

As part of future development on mental health in the Andean population, prospective study designs should be implemented to track and analyze changes in mental health problems over time, both during the course of the pandemic and after it has subsided. This will provide valuable information on the long-term impact and consequences of the pandemic on mental health. In addition, multicenter studies in Latin America will allow for a more representative understanding and assessment of the impact of mental illness on the quality of life of vulnerable rural populations, taking into account the specific challenges and circumstances faced by people in these areas and driving better interventions, support systems, and general wellbeing for these communities.

## Data availability statement

The raw data supporting the conclusions of this article will be made available by the authors, without undue reservation.

## Author contributions

JM-S, BC, and JM-E completed raw data collection and processing. JM-S, AJ-Q, BC, and HC-P performed the data analysis. JM-S, JM-E, and HC-P wrote and edited the manuscript with input from BC. All authors contributed to writing and finalizing the survey questions and to equally distributing the survey and they approved the final manuscript.

## Supplementary material

Supplementary Figure 1 Bias risk assessment of selected studies.
