# Supplementary figures and images for: A systematic review of mental health in rural Andean populations in Latin America during the COVID-19 pandemic
Source: Front Psychiatry. 2023 Aug 17;14:1136328. doi: 10.3389/fpsyt.2023.1136328 (PMC10470633; doi:10.3389/fpsyt.2023.1136328)

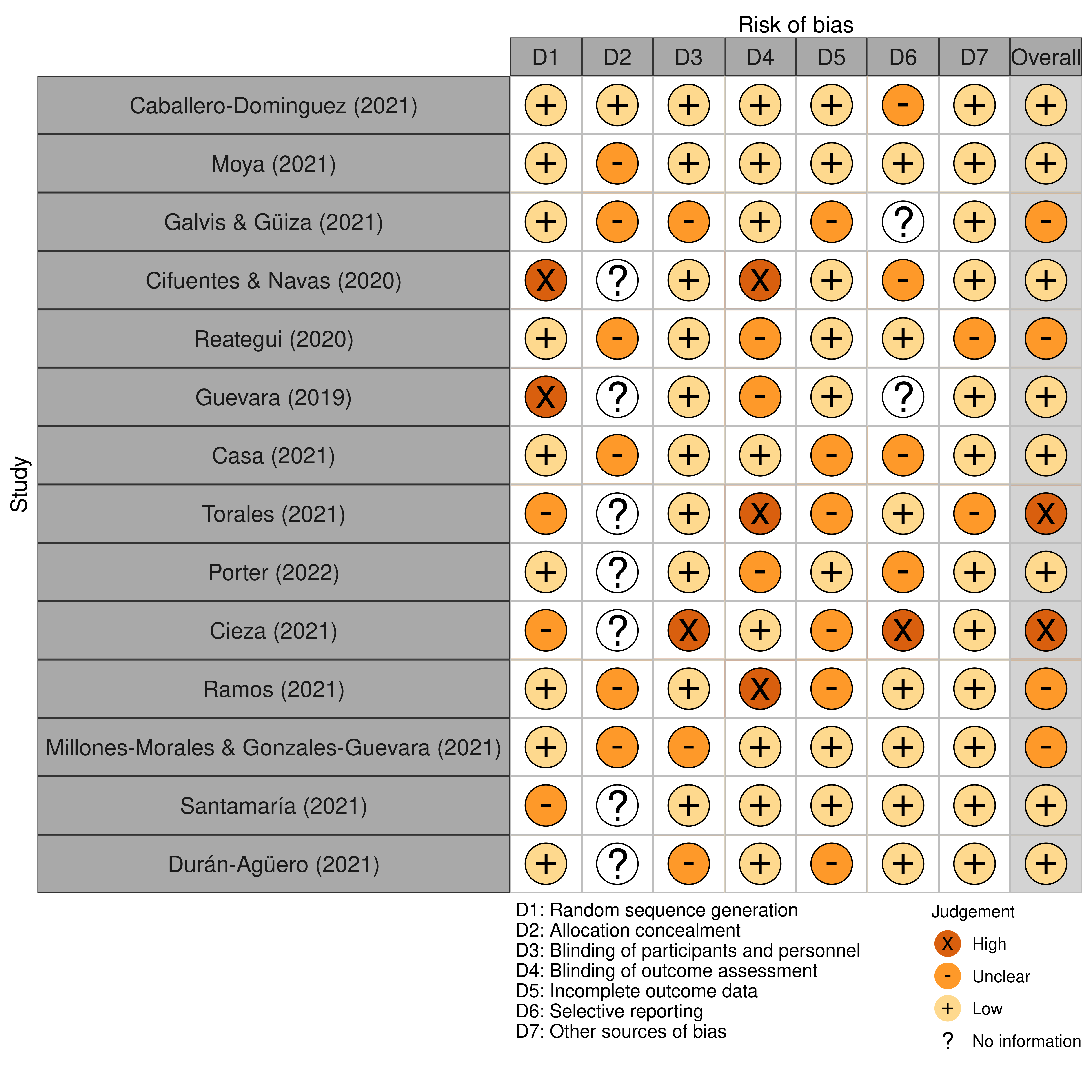

Supplement: Supplementary Figure 1 — Bias risk assessment of selected studies. [file Image_1.TIFF]
